# Editorial

**Published:** 1867-06

**Authors:** 


We give so much of our space in the present number to the Proceedings of the American Medical Association, and the Convention of Delegates from Medical Colleges, that we have none left for book notices and editorial comments.

---

Personal.—Our colleague, Edmund Andrews, M.D., Professor of Surgery in Chicago Medical College, and of Clinical Surgery in Mercy Hospital, will leave for Europe between the 5th and 10th of June. Prof. Andrews goes abroad neither for health or recreation, but for the purpose of profiting by a tour of observation in the hospitals and medical institutions of Europe. He is also a delegate from the American Medical Association to the International Medical Congress, to be held in Paris next. He expects to arrive home again before the 1st of October. We wish him a pleasant and profitable journey and a safe return.

---

Bellevue Place.—This is the name of a private asylum for the treatment of the insane. An advertisement of it will be found in the proper place, in this number of the Examiner. We take special pleasure in calling the attention of our readers to the institution, because we are personally acquainted with Dr. R. J. Patterson, the Superintendent, and regard him as one of the most experienced and skilful managers of the insane in our country, and a man of perfect integrity.

---

Money Receipts from April 22d to May 27th.—Drs. H. H. Guthrie, St. Charles, Minn., $3; M. A. Fox, Shulsburgh, Wis., 3; J. B. LeBland, Brownsville, Minn., 3; A. Grætinger, Milwaukee, Wis., 3; Darius Mason, Prairie du Chien, Wis., 3; Harrison Rodebough, Greenup, Ill., 3; E. L. Holmes, Chicago, Ill., 3; D. B. Bobb, Dakota, Ill., 3; G. W. Rohr, Rockford, Ill, 3; R. L. Walston, Paris, Ill., 3; D. S. Jenks, Plano, Ill., 3; G. M. Fox, Lyons, Ill., 9; D. B. Wren, Fair Haven, Minn., 3; F. F. Stair, Paoli, Wis., 2.50; W. H. Cook, Hillsboro, Ill., 3; E. Dyson, Chicago, Ill., 3; Wm. J. Wheeler, Youngstown, Ohio, 3; E. Winchester, Elgin, Ill., 3.

### Mortality Report for the Month of April:—

CAUSES OF DEATH.

| | | | |
|---|---|---|---|
| Accidents, | 3 | Fever, Scarlet, | 7 |
| Apoplexy, | 2 | Fever, Typhoid, | 12 |
| Bronchitis, | 1 | Fever, not stated, | 3 |
| Burned, | 1 | Gravel, | 1 |
| Cancer, | 2 | Hydrocephalus, | 6 |
| Cold, | 2 | Hæmorrhage, | 1 |
| Croup, | 10 | Inflammation of Bowels, | 3 |
| Cholera Infantum, | 5 | Inflammation of Brain, | 8 |
| Consumption, | 28 | Inflammation of Lungs, | 6 |
| Convulsions, | 20 | Jaundice, | 1 |
| Childbirth, | 4 | Killed, | 4 |
| Congestion of Brain, | 4 | Measlcs, | 1 |
| Congestion of Lungs, | 1 | Old Age, | 8 |
| Congestion, | 1 | Poisoning, | 2 |
| Delirium Tremens, | 2 | Paralysis, | 1 |
| Decline, | 2 | Pneumonia, | 6 |
| Diarrhœa, | 2 | Phthisis Pulmonalis, | 3 |
| Diphtheria, | 8 | Rheumatism, | 1 |
| Dropsy, | 11 | Suffocation, | 1 |
| Drowned, | 8 | Suicide, | 2 |
| Disease of Heart, | 5 | Spinal, | 3 |
| Disease of Liver, | 1 | Stillborn, | 16 |
| Disease of Throat, | 1 | Small-Pox, | 8 |
| Epilepsy, | 1 | Chicken-Pox, | 1 |
| Fever, Brain, | 5 | Spasms, | 1 |
| Fever, Spotted, | 1 | Teething, | 6 |
| Fever, Puerperal, | 1 | Whooping-Cough, | 4 |
| Fever, Lungs, | 10 | Unknown, | 18 |
| Fever, Nervous, | 2 | | |

Total, 278
Total number last year for the month of April, 278

Total number during the month of March, 280
Total number during the month of April, 278

Decrease, 2

DIVISIONS OF THE CITY.

North, 76 | South, 87 | West, 113 | Total, 278
Unknown, 2

Ages of the Deceased.—Under 5 years, 133; over 5 and under 10 years, 9; over 10 and under 20, 13; over 20 and under 30, 26; over 30 and under 40, 30; over 40 and under 50, 23; over 50 and under 60, 13; over 60 and under 70, 11; over 70 and under 80, 7; over 80 and under 90, 1; unknown, 2. Total, 278.

| | | | | | |
|---|---|---|---|---|---|
| Chicago, | 143 | Germany, | 27 | Wales, | 1 |
| Other States, | 40 | Ireland, | 38 | Unknown, | 5 |
| Bohemian, | 2 | Norway, | 1 | | |
| Canada, | 3 | Sweden, | 2 | Total, | 278 |
| England, | 10 | Scotland, | 2 | | |
| France, | 2 | Switzerland, | 2 | | |

